# Anti-Migration and Invasion Effects of Astaxanthin against A172 Human Glioblastoma Cell Line

**DOI:** 10.31557/APJCP.2020.21.7.2029

**Published:** 2020-07

**Authors:** Tanapan Siangcham, Pornpun Vivithanaporn, Kant Sangpairoj

**Affiliations:** 1 *Faculty of Allied Science, Burapha University, Chonburi, Thailand. *; 2 *Chakri Naruebodindra Medical Institute, Faculty of Medicine Ramathibodi Hospital, Mahidol University, Samut Prakan, Thailand. *; 3 *Division of Anatomy, Department of Preclinical Sciences, Faculty of Medicine, Thammasat University, Pathumthani, Thailand. *; 4 *Thammasat University Research Unit in Nutraceuticals and Food Safety, Faculty of Medicine, Thammasat University, Pathumthani, Thailand. *

**Keywords:** Astaxanthin, glioblastoma, migration, invasion, matrix metalloproteinase

## Abstract

**Objectives:**

The study was to investigate anti-migration and invasion effects of astaxanthin (ATX), a natural carotenoid derivative distributed in marine environments, against A172 human glioblastoma cells.

**Materials and Methods:**

Cell viability after ATX treatment was measured by MTT assays. Tumor cell migration and invasion were observed by scratch and Boyden chamber assays, respectively. Expression of MMP-2 and activity of MMP-9 were observed by immunoblotting and gelatin zymography, respectively.

**Results:**

ATX up to 150 µM was not toxic to A172 cells at 48 h post-treatment. In contrast, ATX at 50 and 100 µM significantly decreased migration and invasion of A172 cells at 24 and 48 h post-treatment. Metastatic-reducing effect of ATX is associated with the reduction of MMP-2 and MMP-9 expressions in a dose-dependent manner. Conclusion: This finding indicated that ATX has anti-migration and invasion effects against human glioblastoma cells and might be applicable for the protection against metastasis of glioblastoma.

## Introduction

Glioblastoma (GBM) is an aggressive brain cancer with malignant characteristics. It is the most prevalent and lethal brain cancer. Effectiveness of concurrent surgical resection with chemotherapy for treatment of GBM are still limited (Mooney et al., 2019). Investigation of anti-tumorigenic effects of novel bioactive compounds with low toxicity derived from natural sources that could be produced in a large-scale platform, especially algae, could be beneficial as a food supplement or drug adjuvant.

Astaxanthin (ATX) is a carotenoid derivative, naturally distributed in marine environments as a red pigment. It has been identified in several microorganisms including the microalgae, red yeast, and marine bacterium (Yuan et al., 2011). ATX is considered as a potent antioxidant by its presence of the keto group, which served its beneficial role on disease protection: inflammatory, metabolic, and neurodegenerative diseases relating to oxidative stress (Higuera-Ciapara et al., 2006). Anti-cancer effects of ATX are attributed to various cancer cells such as oral, bladder, colon, leukemia, and hepatocellular carcinomas (Yuan et al., 2011). ATX is beneficial in decreasing cancer progression due to its antioxidant properties, which mediate through a variety of mechanisms, including inflammation, cell interaction, and cell death (Zhang and Wang, 2015). 

Migration and invasion are crucial cellular mechanisms of tumor metastasis. This process is mainly regulated by matrix metalloproteinases (MMPs), a major group of tissue-degrading enzymes (Curran and Murray, 2000; Mentlein et al., 2012). MMPs are zinc-dependent endopeptidases produced from tumor cells to degrade the ECM as the target (Mentlein et al., 2012). Disturbance in this process eliminates cancer progression and is widely focused as a therapeutic target. ATX treatment at 10 to 50 µM for 24 h decreased proliferation and reduced migration of breast cancer cells with no apparent effect on apoptotic marker expression (McCall et al., 2018). This study presented anti-metastatic effects of ATX against A172 human glioblastoma cells by inhibiting migration and invasion abilities with reducing MMP-2 and MMP-9 expressions as target molecules.

## Materials and Methods

*Cell culture and reagents*

A172 Human glioblastoma cell lines, obtained from ATCC (USA) were cultured in DMEM containing 10% FBS in a cell incubator with an atmosphere of 5% CO_2_ at 37°C. Astaxanthin (ATX) purified from Haematococcus pluvialis (Sigma-Aldrich, USA) is dissolved in dimethyl sulfoxide (DMSO) then heated at 40°C for 10 min before use.

*Treatment and cytotoxicity test*

A172 cells cultured in 96-well plates were treated with increasing concentrations of ATX diluted in serum-free media for 48 h. The viability of treated cells was tested by MTT (Sigma, USA) at a final concentration of 0.4 mg/ml, followed by 3 h incubation and measured by spectrophotometer at the absorbance of 562 and 630 nm (Flow Laboratories, VA, USA). Percentage of cell viability was calculated following the formula:

% viability = A_test_/A_control_ ×100

(A=A_(562 nm)_-A_(630 nm)_)

*Tumor migration assay*

Cell migration following ATX incubation was determined by wound scratch assays. Wound was created by scratching of 200 µl pipette tip on confluent cultured cells followed by ATX treatment at 50 and 100 µM. The gap width was observed at 0.24, and 48 h under a Nikon Ds-Ri1 inverted microscope (Nikon, Japan) at 4 x magnification. The width of the wound area was measured by ImageJ software (http://rsb.info.nih.gov/ij/).

*Tumor invasion assay*

Invasive ability was tested using Boyden chamber assays with modified two chambers containing 8 µm pore size. Invasion insert chambers were coated with Matrigel (Corning, NY, USA) in serum-free media. A172 cells were treated with ATX at 50 and 100 µM then seeded into the upper chambers with serum-free media. The serum-containing media were added to the lower chamber. After incubation for 48 h at 37°C, non-invasive cells at the top of the upper chamber were gently removed by a cotton swab. Invasive cells passing through Matrigel at the bottom of the upper chamber were fixed with 4% (w/v) paraformaldehyde and stained with crystal violet. Invasive cells were visualized under a Nikon Ds-Ri1 inverted microscope (Nikon, Japan) at 10 x magnification. Stained cells in five fields (upper left/right, median and lower left/right) were counted.

*Immunoblotting*

Whole cellular proteins were extracted using RIPA cell lysis buffer (Cell Signaling Technology). Protein concentrations were determined using Bradford protein assays (Bio-rad, USA). Equivalent amounts of whole proteins (30 µg each) were separated by 15% SDS-PAGE then transferred onto nitrocellulose membranes. The membranes were blocked in Tris-Buffered Saline and Tween-20 (TBST) containing 5% BSA for 1 h then incubated with the primary antibodies at 4°C overnight. The primary antibodies, including rabbit anti-MMP-2 and rabbit anti-β-actin (Cell Signaling Technology) were used. The membranes were washed with TBST then incubated with corresponding HRP-conjugated secondary antibodies (Southern Biotech) diluted in 0.01 M TBST for 1:5,000 dilutions at room temperature for 1 h. Expression of specifically targeted proteins were detected by ECL chemiluminescence system (Thermo Fisher Scientific, Waltham, MA, USA) and visualized by ChemiDoc™ Touch Imaging System (Bio-rad, USA). The relative expression level of MMP-2 protein was normalized to β-actin expression compared with the control group. 

*Gelatin zymography*

Enzyme activity of MMP-9 was analyzed by gelatin zymography. The conditioned media after cell treatment for 24 h was collected and centrifuged to remove cell debris. The supernatant was concentrated with Amicon ultra centrifugal filter 10,000 MWCO (Merck Millipore) by using centrifugation at 4,500g for 30 min until the remaining volume was 100 µl. From the concentrated conditioned media samples, 10 µl was mixed with 4X non-reducing buffer and electrophoresed in 8% SDS-PAGE with 1% gelatin. The gel was placed in the renaturing buffer (2.5% v/v Triton X-100 in distilled water) to remove SDS then incubated with a buffer (50 mM Tris, 0.15 M NaCl, 5 mM CaCl_2_, NaN_3_, pH 7.6) for 48 h at 37°C. Gelatinolytic activity was visualized by staining the gels with Coomassie blue G 250 solution (Sigma) for 1 h then destained in destaining solution (4% methanol, 8% glacial acetic acid). Band intensity was analyzed and semi-quantified by ImageJ analysis software (http://rsb.info.nih.gov/ij/). Band density of the untreated group in each experiment was defined as 100%.

*Statistical analysis*

Data were expressed as mean ± SEM. Statistical variations of all experiments were analyzed by GraphPad Prism statistical analysis software (GraphPad Software Inc, USA) using a one-way ANOVA test. A p-value less than 0.05 was considered statistically significant.

## Results

*Cytotoxicity test*

**Figure 1:**
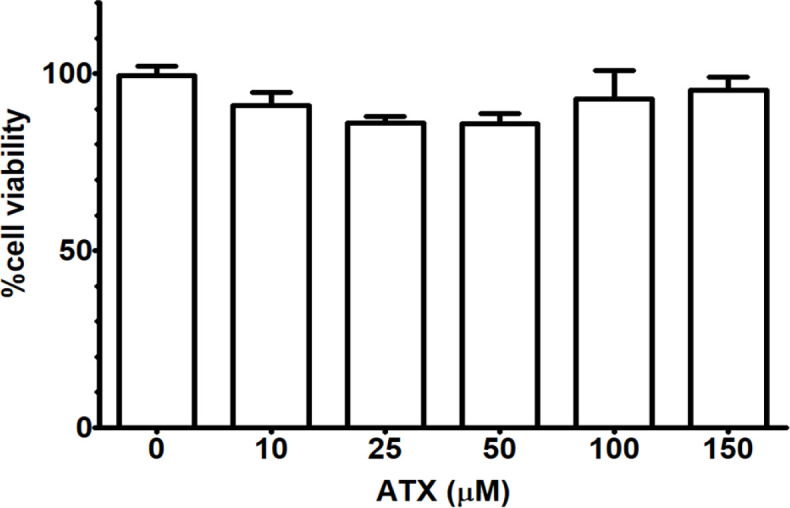
Percentage of Cell Viability of A172 Cells Following ATX Treatment at 48 h

Cytotoxic effect of ATX against A172 human glioblastoma cells was tested using MTT assays following treatment with increasing concentrations of ATX for 48 h. The viability of A172 cells with ATX up to 150 µM was similar to the control group at all selected concentrations (Figure 1). These results indicated that ATX is not toxic to A172 cells in a time- and concentration-dependent manner. 

*Tumor cell migration*

**Figure 2:**
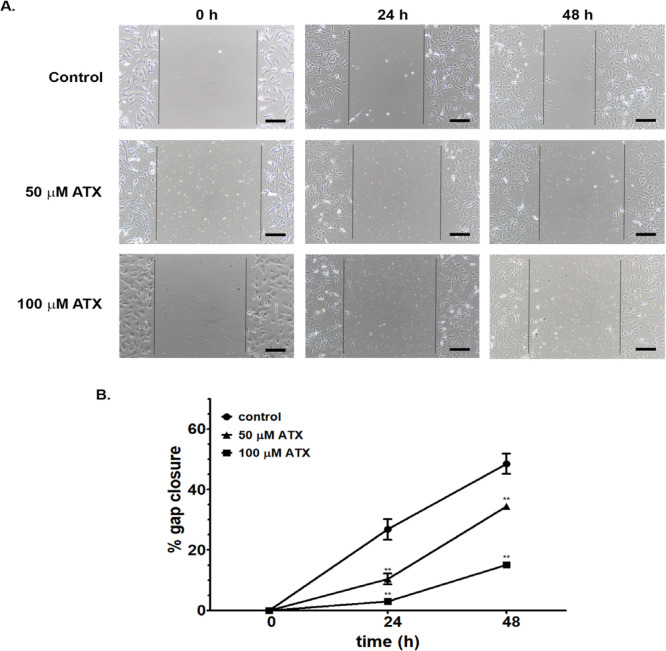
Migration of A172 Cells Following ATX Incubation at 50 and 100 µM for 24 and 48 h. (A) The photomicrographs of migrating cells were shown at a low magnification (Scale bar = 200 µm). The vertical lines indicating the initial scratch boundary at 0 h and migrating fronts at 24 and 48 h. The migration ability indicated as the percentage of gap closure on cultured surface. (B) The percentage of gap closure after ATX exposure compared with the control group of each time point. ** p ≤ 0.01

The effect of ATX on tumor cell migration was investigated by scratch assays. Migration ability of A172 cells was observed following ATX treatment at 50 and 100 µM for 24 and 48 h. Both selected concentrations of ATX could decrease migration of A172 cells. The percentages of gap closure after treatment with both concentrations of ATX at 24 and 48 h were significantly lower than that of the control group (Figure 2). ATX at 100 µM decreased migration of A172 cells more than at 50 µM. This result suggested that ATX inhibited the in vitro migration of A172 cells.

*Tumor cell invasion*

**Figure 3:**
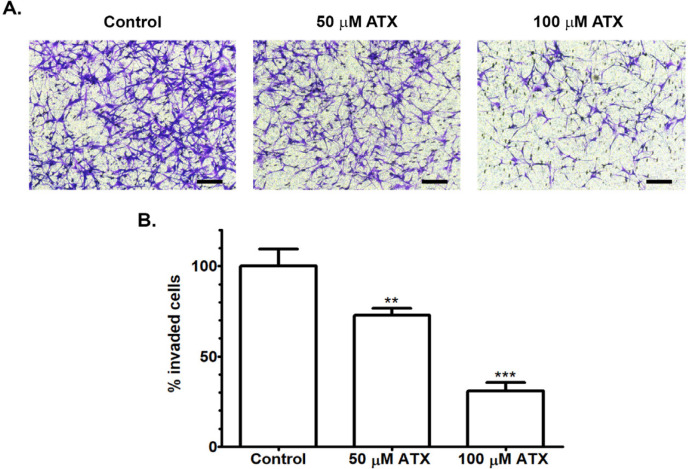
Reduction of A172 Cell Invasion after Incubation with ATX for 48 h. (A) The photomicrographs of invading cells through the Boyden chamber were shown at a higher magnification (Scale bar = 50 µm). (B) The number of invading cells were estimated as the percentage of invasion compared with the control group. ** *p *≤ 0.01, *** *p *≤ 0.001

Effect of ATX on regulating tumor cell invasion was observed by Boyden chamber assays. The invasiveness of A172 cells is determined by counting the number of invaded A172 cells through a permeable membrane coated with an extracellular matrix. Treatment of ATX at both concentrations reduced the invasive ability of A172 cells, as shown by lowering the percentage of invaded cells compared with the untreated group (Figure 3). This result indicated that ATX inhibited the invasion of GBM cells.

*Expression of invasive-related proteins following ATX treatment*

**Figure 4:**
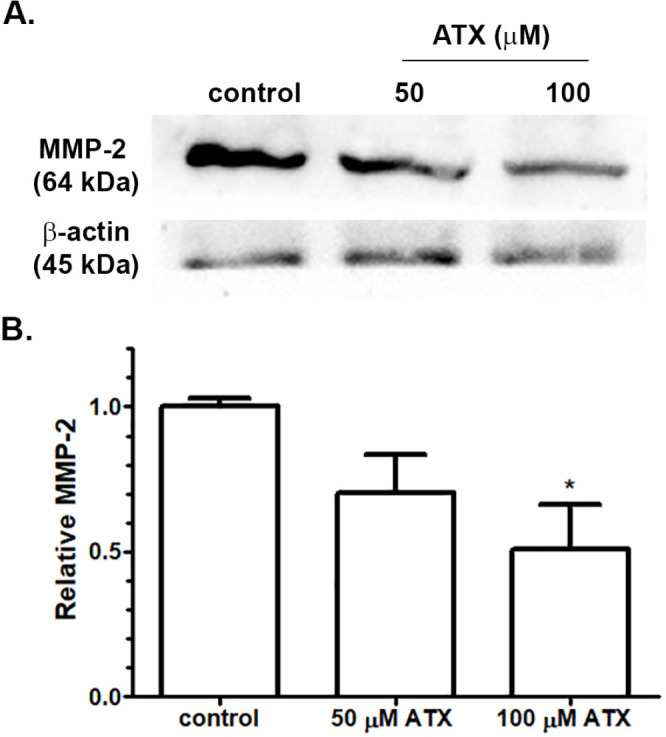
(A) The expression of MMP-2 protein following ATX treatment in A172 cells was analyzed by immunoblotting. (B) Relative expression of MMP-2 after ATX treatment for 24 h compared with the untreated control group. * *p *≤ 0.05

**Figure 5:**
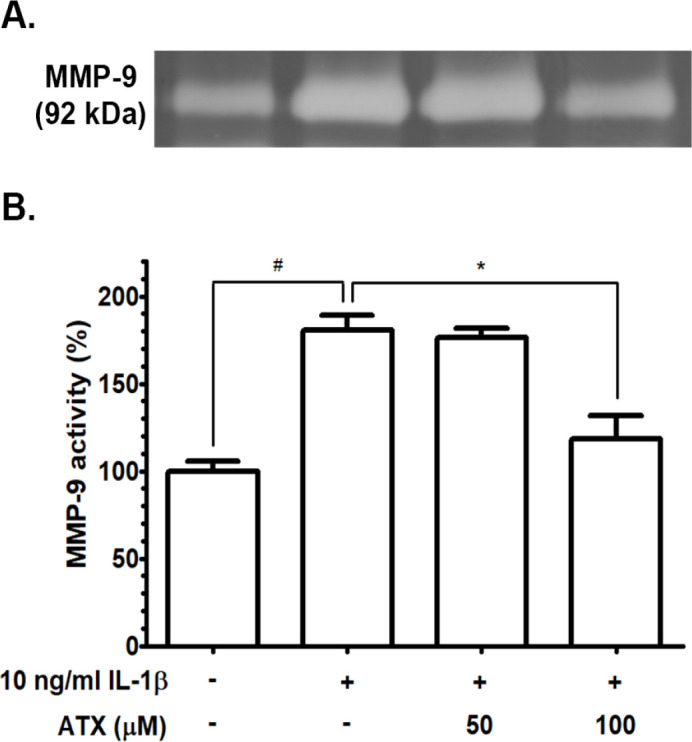
(A) The enzyme activity of MMP-9 after ATX treatment for 24 h assayed by gelatin zymography. (B) The percentage of MMP-9 activity of IL-1β-induced A172 cells for 24 h compared with untreated group (# p ≤ 0.05) and IL-1β-induced cells compared with IL-1β and ATX-treated cells (* *p* ≤ 0.05).

Expression levels of MMP-2 and MMP-9, major metalloproteinase proteins, produced by GBM cells during tumor invasion was observed following ATX incubation for 24 h by immunoblotting and gelatin zymography, respectively. The relative expression level of MMP-2 proteins in A172 protein lysates was significantly decreased by treatments with increasing concentrations of ATX at 50 and 100 µM when compared with the untreated group (Figure 4). Enzymatic activity of MMP-9 was assayed after stimulation with 10 ng/ml IL-1β and ATX incubation. The activity of MMP-9 was evaluated and presented by the increase of the in-gel digestion of gelatin. MMP-9 gelatinolytic activity increased after IL-1β incubation and attenuated after treatments with ATX, especially at 100 µM (Figure 5). These results indicated that ATX downregulated MMP-2 expression and MMP-9 activity in A172 cells.

## Discussion

Degradation of the ECM components are crucial for GBM cell migration and proliferation spreading into the surrounding brain parenchyma. The family of MMPs is implicated as a key enzyme for ECM degradation. In GBM, MMP-2 and MMP-9 are gelatinase enzymes that are highly expressed and associated with its invasiveness (Lakka et al., 2002; Kargiotis et al., 2008). Thus, the reduction of MMP-2 and MMP-9 expression and activity may inhibit GBM invasion. 

ATX is a carotenoid derivative found in microalgae and marine organisms, used primarily as a food supplement. In animal models, ATX has pharmacological properties for protection against breast and colon cancers. Dietary astaxanthin could protect mammary tumor initiation in BALB/c mice by upregulating the immune response through the increase of natural killer cells and plasma interferon-γ when fed before the tumor initiation (Nakao et al., 2010). Pre-treatment of astaxanthin also reduced oxidative stress-induced lipid peroxidation and tumor proliferation against chemically-induced colon cancer in rats (Prabhu et al., 2009). Moreover, ATX exerts a strong neuroprotection since its fat-soluble properties allow it to readily cross the blood-brain barrier (Galasso et al., 2018). It has been suggested that ATX has a potential for neuroprotection of neurological pathologies including Alzheimer’s diseases, Parkinson’s diseases, traumatic brain injury, and age-related dementia (Fakhri et al., 2018). Thus, ATX might be suitable for prevention of GBM metastasis because of its penetrability to the brain as the target site. 

Anti-metastatic effects of ATX have been observed in melanoma and colon cancer cells. ATX down-regulated MMP-1, MMP-2, and MMP-9, resulting in the inhibition of migration and invasion of melanoma cells in a dose-dependent manner from 5 – 125 µg/ml for 24 h (Chen et al., 2017). ATX reduced the metastasis of colon cancer cells by decreasing the expression of MMPs through inhibition of NF-kB and COX-2 transcription factors and repressed EMT formation through the increase of miR-29a-3p and miR-200a expression (Nagendraprabhu and Sudhandiran, 2011; Kim et al., 2019). These previous reports implied that MMP might be the target of ATX treatment for inhibition of tumor metastasis. 

Our results demonstrated that ATX treatment decreased migration and invasion of A172 cells in a time- and concentration-dependent manner. These effects were correlated with the decrease of MMP-2 and MMP-9 expressions following ATX incubation in a concentration-dependent manner. Thus, this recent finding indicated that ATX could inhibit migration and invasion of A172 cells through down-regulation of MMP-2 and MMP-9. In addition, ATX was not toxic to A172 cells. This implied that the anti-invasive effect of ATX in GBM is not mediated by growth–inhibitory effect. 

In conclusion, ATX significantly decreased migration and invasion abilities of A172 human GBM cells in a time- and concentration-dependent manner. This effect is correlated with down-regulation of matrix degrading enzymes, MMP-2 and MMP-9. This recent finding suggested that ATX might be a potential natural compound for protecting GBM progression by suppressing its invasion. Molecular mechanisms underlying ATX activity for GBM prevention would be further investigated.
